# Effect of Inoculum Size and Age, and Sucrose Concentration on Cell Growth to Promote Metabolites Production in Cultured *Taraxacum officinale* (Weber) Cells

**DOI:** 10.3390/plants12051116

**Published:** 2023-03-02

**Authors:** María Eugenia Martínez, Lorena Jorquera, Paola Poirrier, Katy Díaz, Rolando Chamy

**Affiliations:** 1 Escuela de Ingeniería Bioquímica, Facultad de Ingeniería, Pontificia Universidad Católica de Valparaíso, Brasil 2085, Valparaíso 237463, Chile; 2 Escuela de Ingeniería en Construcción y Transporte, Facultad de Ingeniería, Pontificia Universidad Católica de Valparaíso, Avenida Brasil 2147, Valparaíso 237463, Chile; 3 Departamento de Química, Universidad Técnica Federico Santa María, Avenida España #1680, Valparaíso 2390123, Chile

**Keywords:** dandelion, triterpene, secondary metabolite, cell suspension, lupeol, α-amyrin

## Abstract

Pentacyclic triterpenes, including lupeol, α- amyrin, and β-amyrin, present a large range of biological activities including anti-inflammatory, anti-cancer, and gastroprotective properties. The phytochemistry of dandelion (*Taraxacum officinale*) tissues has been widely described. Plant biotechnology offers an alternative for secondary metabolite production and several active plant ingredients are already synthesized through in vitro cultures. This study aimed to establish a suitable protocol for cell growth and to determine the accumulation of α-amyrin and lupeol in cell suspension cultures of *T. officinale* under different culture conditions. To this end, inoculum density (0.2% to 8% (*w*/*v*)), inoculum age (2- to 10-week-old), and carbon source concentration (1%, 2.3%, 3.2%, and 5.5% (*w*/*v*)) were investigated. Hypocotyl explants of *T. officinale* were used for callus induction. Age, size, and sucrose concentrations were statistically significant in cell growth (fresh and dry weight), cell quality (aggregation, differentiation, viability), and triterpenes yield. The best conditions for establishing a suspension culture were achieved by using a 6-week-old callus at 4% (*w*/*v*) and 1% (*w*/*v*) of sucrose concentration. Results indicate that 0.04 (±0.02) α-amyrin and 0.03 (±0.01) mg/g lupeol can be obtained in suspension culture under these starting conditions at the 8th week of culture. The results of the present study provide a backdrop for future studies in which an elicitor could be incorporated to increase the large-scale production of α-amyrin and lupeol from *T. officinale*.

## 1. Introduction

Dandelion (*Taraxacum* sp.) has been used extensively as a traditional medicine for hundreds of years, primarily due to its medicinal properties, such as anti-inflammatory, anti-carcinogenic, and anti-rheumatic effects, among others. Medicinal plants typically contain several different chemical compounds that may act individually, additively, or in synergy to improve pharmacological properties. Several compounds have been identified from various organs of the dandelion plant to which medicinal activity has been attributed, primarily comprising various triterpenes (α-/β-amyrin, lupeol, taraxerol, taraxasterol, amidiol, faradiol), sterols, and phenolics (caffeic acid, phenylacetic acid, and chlorogenic acids) [1,2,3]. Specifically, pentacyclic triterpenes, including lupeol, α-amyrin, and β-amyrin, present a large range of biological activities including anti-inflammatory, antioxidant, anti-carcinogenic, and gastroprotective properties [4,5,6]; these triterpenes have been widely reported in *Taraxacum*’s tissues. *T. officinale* has great potential applicability as a food, cosmetic, and medicine due to its phytochemical characteristics supported by pharmacological research [7]. Plant biotechnology offers an alternative, and several active plant ingredients are already synthesized through in vitro cultures [8].

In vitro propagation methods are widely used for plants with cultivation difficulties and/or low yields and/or productivities, or if technical problems arise [9]. Among in vitro cultures, callus and plant cell suspension cultures provide rapid plant multiplication and allow manipulation of the environment, either to produce specific compounds or to increase their yields [10]. Specifically, the suspension culture is considered a stable production platform, ensuring consecutive production of natural products of uniform quality and yield, providing homogeneity and higher cell propagation efficiency compared to callus cultures grown on solidified medium in parallel [11]. From an engineering perspective, suspension culture has more immediate potential for industrial applications than callus or organ cultures [12]. In comparison with whole-plant systems, cells in suspension have a relatively shorter growth life cycle and remain undifferentiated, providing a continuous supply of experimental units grown under controlled environmental conditions [13]. Nevertheless, there are still unavoidable problems, including the instability of cell lines, low productivity, slow growth, and scale-up obstacles, that result in lower production of metabolites [11].

Many factors affect cell growth and secondary metabolites synthesis in the suspension culture, including nutrients and energy sources [14], plant growth regulators [15,16], conditioned medium [17], and ethylene/CO_2_ accumulation [18]. Specifically, for sucrose concentration in suspension cultures, differing outcomes have been reported regarding the effects of sucrose concentration on plant cell cultures. Sucrose not only acts as an external energy source but also contributes to the osmotic potential of the medium. allowing the absorption of mineral nutrients present in the medium, stimulating mitochondrial activity, and, hence, production of the energy required for metabolite synthesis [19,20]. Moreover, many researchers have pointed out the effects of sucrose concentration on secondary metabolite biosynthesis, during which development, chemical profile, and yields of in vitro cell cultures were highly dependent on the type and concentration of carbohydrates used in the medium [21,22,23].

For the *Taraxacum* genus, triterpene accumulation has been previously described for callus culture compared to wild plants [24,25]. Nonetheless, few studies on suspension cultures are available, and they are primarily focused on secondary metabolic studies. Recently, the establishment of an ex vivo laticifer cell suspension culture from *T. brevicorniculatum* as a production system for cis-isoprene was reported [26]. In other work, a hairy suspension culture of *T. officinale* was conducted to evaluate the yield increase of taraxasterol and taraxerol through elicitation by the addition of abiotic elicitors like methyl jasmonate and β-cyclodextrin [27]. This is the first work that characterizes the growth response of *T. officinale* suspension culture under different culture conditions to determine the quantification of lupeol and α-amyrin. Both molecules were selected in this study because they are metabolites of high pharmacological and commercial value and previous reports from the same research team reported that both secondary metabolites (lupeol and α-amyrin) are present in the leaves of wild plants in the *Taraxacum* species in a more abundant amount than other compounds of the triterpene group [28]. For this reason, this study aims to establish a suitable protocol for cell growth and to evaluate the effect of different inoculum conditions (age and size) and sucrose concentrations on the accumulation of triterpenes (α-amyrin and lupeol) in cell suspension cultures of *T. officinale*.

## 2. Results

### 2.1. Effect of Inoculum Age and Size on Cell Suspension Culture Establishment

#### 2.1.1. Cell Growth and Viability

A second-order polynomial model of dependent comprehensive responses on coded independent variables (Age and Size of inoculum) was established following a nonlinear regression technique for cell growth and viability. The estimation of dry weight, fresh weight, and viability under the proposed conditions could be modeled by Equation (1), Equation (2), and Equation (3), respectively; where A: Age and B: Size. (See Section 4.4).

(1) $$X\;\left(\frac{g\;\mathrm{dw}}{L}\right)=-3.15+3.40\cdot B-0.11\cdot A^{2}-0.55\cdot B^{2}$$

(2) $$X\;\left(\frac{g\;\mathrm{fw}}{L}\right)=-115.64+110.58\cdot B-4.23\cdot A^{2}-16.56\cdot B^{2}$$

(3) $$\mathrm{Viability}\;\left(\%\right)=62.9+4.31\cdot A+12.31\cdot B-0.31\cdot A^{2}-1.81\cdot B^{2}$$

Regression and variance analysis were applied to fit the model and to assess the statistical significance of the terms for cell growth and viability. The analysis of variance of the polynomial models for the response variables, along with the corresponding R-Squared, Adj R-Squared, Pred R-Squared, C.V., and Adeq Precision, is shown in Table 1, Table 2, and Table 3 for dry weight, fresh weight, and viability, respectively.

For cell dry weight, the Model F-value of 56.59 implies the model is significant. In this case, B, A^2^, B^2^ are significant model terms. The “Lack of Fit F-value” of 8.17 implies the Lack of Fit is significant relative to the pure error. There is a 0.67% chance that a “Lack of Fit F-value” this large could occur due to noise. This demands another set of experiments for model validation in the future. The “Pred R-Squared” of 0.9044 is in reasonable agreement with the “Adj R-Squared” of 0.9392. “Adeq Precision” measures the signal-to-noise ratio. Because a ratio greater than 4 is desirable, a ratio of 20.034 indicates an adequate signal. Therefore, this model can be used to navigate the design space.

For cell fresh weight, the Model F-value of 15.74 implies the model is significant. In this case, B, A^2^, B^2^ are significant model terms. The “Lack of Fit F-value” of 1.31 implies the Lack of Fit is not significant relative to the pure error. There is a 30.80% chance that a “Lack of Fit F-value” this large could occur due to noise. The “Pred R-Squared” of 0.7217 is in reasonable agreement with the “Adj R-Squared” of 0.8037. “Adeq Precision” measures the signal-to-noise ratio. Because a ratio greater than 4 is desirable, a ratio of 10.205 indicates an adequate signal. Therefore, this model can be used to navigate the design space.

The Model F-value of 23.59 implies the model is significant. In this case, A, B, A^2^, B^2^ are significant model terms. The “Lack of Fit F-value” of 2.69 implies the Lack of Fit is not significant relative to the pure error. There is an 11.20% chance that a “Lack of Fit F-value” this large could occur due to noise. The “Pred R-Squared” of 0.7642 is in reasonable agreement with the “Adj R-Squared” of 0.8714. “Adeq Precision” measures the signal-to-noise ratio. Because a ratio greater than 4 is desirable, a ratio of 13.496 indicates an adequate signal. Therefore, this model can be used to navigate the design space.

**Figure 1:**
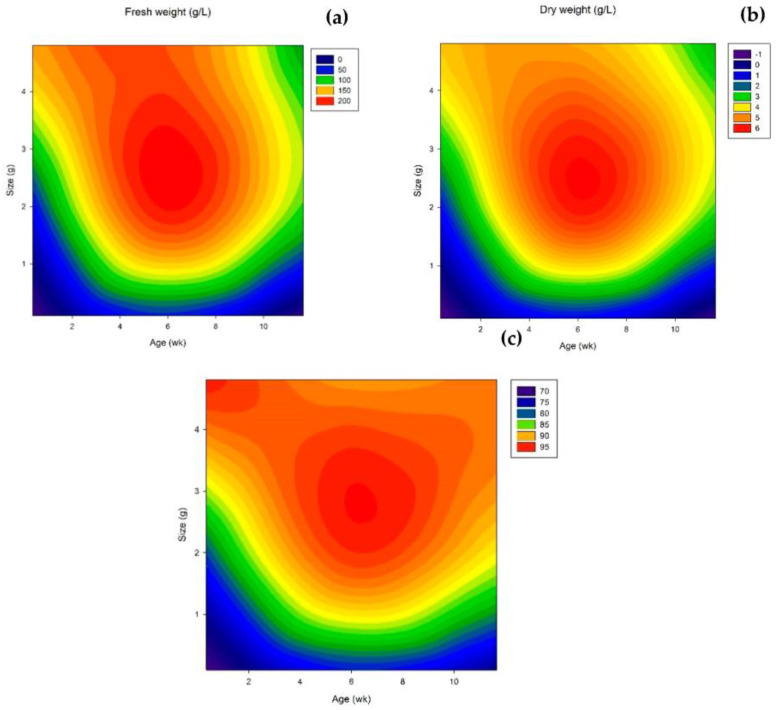
Response surface contour plots showing the interaction effects of inoculum age and size on cell suspension culture on (**a**) Fresh weight, (**b**) dry weight, (**c**) viability of the suspension cultures of *T. officinale* inoculated with callus at different ages (2- to 10-week-old) and sizes (1% to 40% (*w*/*v*)) (Basal MS medium, sucrose 2.3% (*w*/*v*), 3.0 mg/L NAA, 3.0 mg/L BAP, 20 ± 2 °C, 115 rpm). The blue color indicates the least optimal values of each variable, while the interaction value approaches the red color, the higher the response variable.

For the three parameters evaluated (dry and fresh weight and viability), statistical analysis showed that is only a 0.01% chance (*p* < 0.0001) that a “Model F-Value” this large could occur due to noise, while values of “Prob > F” less than 0.0500 indicate model terms are significant. Also, R^2^ values, Adj R^2^ values, and Pred R^2^ values were satisfactory, implying that a high percentage of response variations were explained by the correspondent response surface equation. In Figure 1a,c, the comprehensive effect of cell growth (fresh and dry weight) and viability of the suspension cultures inoculated with callus at different ages (2- to 10-week-old) and sizes (0.2% to 8% (*w*/*v*)) could be further represented with the response surface plots as presented. These variables were found significant in cell growth when fresh and dry weights were assessed (Table 1 and Table 2).

Fresh and dry weight was found to be higher when 20–40% *w*/*v* of 4–8-week-old calli were used for initiating the suspension culture. Results showed that the highest biomass accumulation in the suspension was 204.4 g fw/L (6.0 g dw/L with 94% viability) when 20% *w*/*v* of 6-week-old calli were used as inoculum to initiate the cell suspension culture, while the lowest values were obtained when a small amount (<10% (*w*/*v*)) of young cultures (2-week-old) was used.

In general, viability was considered adequate for the suspension cultures of *T. officinale*, especially at the end of the experimental period. However, when 2- and 10-week-old calli were used, viability was lower than 80% when small-size inoculum (<10% (*w*/*v*)) was used, presenting a high amount of debris floating in the suspension medium, often forming clumps with the bigger cell clusters.

#### 2.1.2. Cell Aggregation and Differentiation

General observations during the first month showed that Group 1 (small cluster, <5 cells/aggregate) was somehow higher in suspensions started with small inoculums while Group 3 (large cluster, >25 cells/aggregate) was preponderant when big cell masses were used as inoculums. However, this tendency derived from the initial cell disaggregation from the callus surface due to mechanical forces through flask agitation, and no statistical significance was observed (*p* > 0.05). Nonetheless, a weak tendency (R^2^ = 0.41) was observed for Group 2 (625 cells/aggregate) in a 2FI Model presenting a local maximum at the border conditions of low age and size (data not shown). After two months of the suspension culture initiation, aggregation in Group 2 was largest (near 60%) in cultures started with 0.2% (*w*/*v*) of a 2-week-old inoculum. Throughout the entire experimental period, no organogenesis was observed in the suspension culture.

Regarding viability, a trypan blue test proved to be sufficient and complementary to morphological changes to indicate the viability of the culture during the experiments, which was higher than 75% throughout. However, an important quantity of debris was observed, especially at the beginning of the experiment, in which cells were trapped (especially during the first subculture), probably derived directly from the callus surface during agitation during this first month. Even when debris was removed from the 2nd month, initial growth and cell adaptation could be affected by the presence of these residues.

Based on the best results obtained in experiment 1, the conditions for experiment 2 were established. Therefore, suspension cell culture was carried out using 4% (*w*/*v*) of a 6-wk old friable callus as inoculum, that was maintained under CM medium. The culture was maintained for 2 months before the sucrose concentration was changed and evaluated. This suspension culture was able to maintain medium clusters (625 cell/aggregate) and viability above 60% and 95%, respectively, during the entire period.

**Figure 2:**
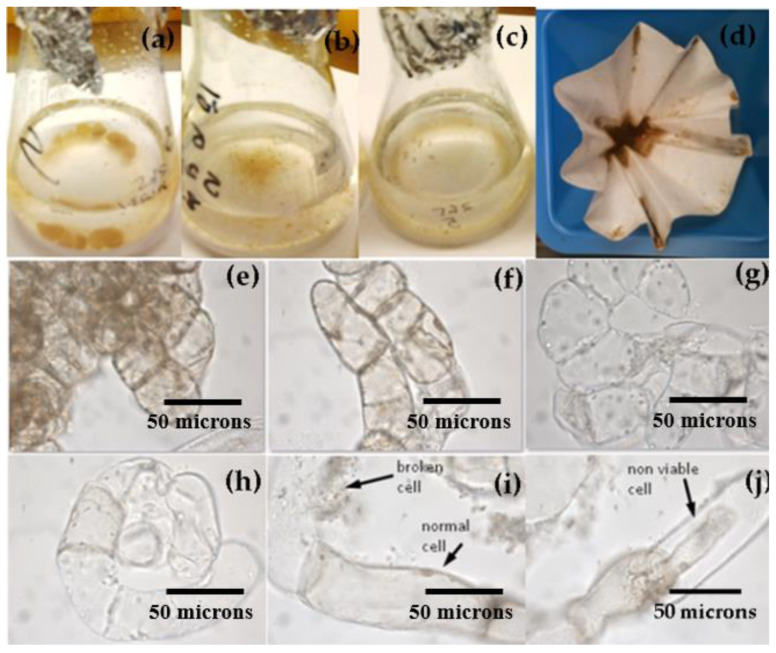
Suspension culture of *T. officinale* during the entire experimental period. *T. officinale* suspension culture. Suspension cultures at (**a**,**e**) 2nd week, (**b**,**f**) 4th week, and (**c**,**d**,**g**,**h**) 8th week, (**i**) Normal, broken cells, (**j**) non-viable cells. (Basal MS medium, 2.3% *w*/*v* sucrose, 3.0 mg/L NAA, 3.0 mg/L BAP, 0/24h light/dark, 20 ± 2 °C, 115 rpm, at 16th week of culture). Bars = 50 microns.

In Figure 2, suspension cultures of *T. officinale* are shown. For each of the treatments, cell suspension cultures initiated from calli at the previously selected conditions (4% (*w*/*v*) of 6-week-old calli) consisted initially of big pale-yellow/white cellular masses. After periodical filtrations (every week from the 4th week of culture), the biomass was finally composed of a mixture of fine cells and clusters of different sizes (Figure 2a–d). The microscopic observation indicated that cells changed their shape during the experimental period. Young cultures presented spherical shapes (Figure 2e–g) before elongation occured from the 4th–5th week of maintenance (Figure 2h,i). From the 8th week, cells were more elongated, showing a concatenated aspect, after which biomass primarily consisted of chains of different lengths in the cultures at 1, 2.3, and 3.2 (*w*/*v*) of sucrose (Figure 2j). At 5.5% (*w*/*v*) of sucrose, cells turned highly compact in their cytoplasm and presented a dark brown color. However, viability remained above 75% for all the conditions tested.

In Table 4, quality parameters of viability, differentiation, and aggregation during the experiment are presented. Excepting differentiation, these parameters were indeed affected by the carbon source concentration, showing less necrotic cells and smaller aggregates (especially for Group 2 or medium clusters, 6–25 cells/aggregate) at lower carbon source concentrations (1.0 and 2.3 (*w*/*v*)) (*p* < 0.001). Viability was similar for the suspension cultures maintained at 1.0%, 2.3%, and 3.2% (*w*/*v*) of sucrose, with values of 87%, 91%, and 87%, respectively. Slightly lower water content was determined in the cultures maintained at 5.5% (*w*/*v*) of sucrose, presenting an average value of 79%. No differentiation was observed during the experimental period.

Finally, the pH of the growth medium decreased slightly from 5.8 to 5.5 during the first months of the culture. When exponential growth initiated (approx. from the 5th week), pH variation between cycles became higher, decreasing to values in the range of 4.9–5.2 at the end of the subculturing period, especially for cultures at 1.0% and 2.3% (*w*/*v*) of sucrose (Figure S1, Supplementary material).

#### 2.1.3. Triterpene Content

Results fitted weakly for α-amyrin (R^2^ = 0.53) and lupeol (R^2^ = 0.49) in a linear model related to the age factor when the size factor was 4% (*w*/*v*) g (data not shown). Results indicated higher values when inoculum age was 6 weeks and size was between 4 and 8% (*w*/*v*), with values of 0.09 mg/g for α-amyrin and 0.19 mg/g for lupeol. A ratio of lupeol/α-amyrin of 2.5 was observed for the conditions evaluated.

### 2.2. Effect of Carbon Source on Cell Suspension Cultures

#### 2.2.1. Effect of Carbon Source on Cell Growth

**Figure 3:**
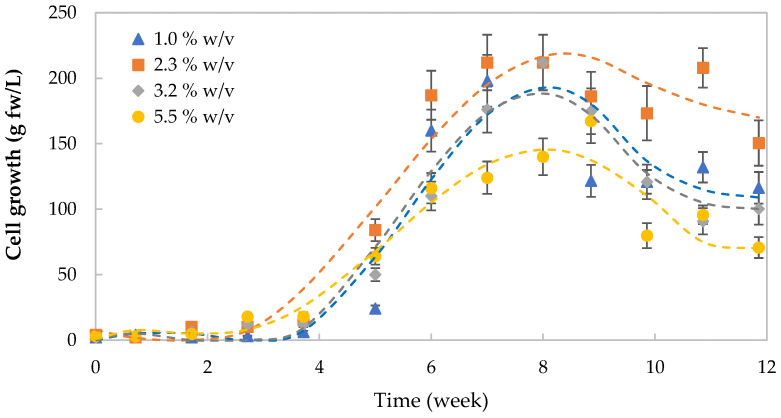
Growth kinetics expressed as dry weight (g/L) of *Taraxacum officinale* cell cultures maintained in different growth media containing 1.0%, 2.3%, 3.2%, and 5.5% (*w*/*v*) sucrose for 12 weeks. Each value is the mean of three replicates.

The cells were grown in culture media with different carbon sources, demonstrating that the maximum biomass production was higher in the 8th week of the medium containing 2.3% (*w*/*v*) sucrose (Figure 3). It should be noted that the cell cultures that generated the lowest biomass were at 1.0% and 5.5% (*w*/*v*) sucrose. It was observed that there was a tendency for the cells to form clusters during the growth kinetics. The exponential growth phase was observed between the 5th and 9th week with a rapid decrease after this point, excepting the culture at 2.3% sucrose. Moreover, the viability of the cells was considerably lower in this period.

**Figure 4:**
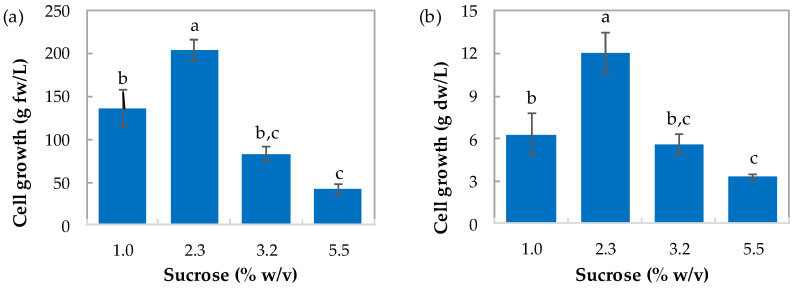

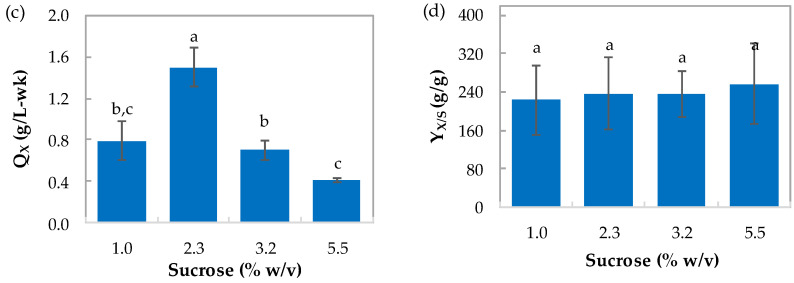
Cell growth parameters: (**a**,**b**) fresh and dry weight, (**c**) biomass yield (Y_X/S_), and (**d**) productivity) (Q_X_) in suspension cultures of *T. officinale* maintained at different sucrose concentrations (1.0, 2.3, 3.2, and 5.5% (*w*/*v*)) (Basal MS medium, 3.0 mg/L NAA, 3.0 mg/L BAP, 20 ± 2 °C, 115 rpm, 8th week of culture). Means with the same letter above the bars were not significantly different at the 0.05 level according to the DMRT test. Bars indicate standard error.

In Figure 4, cell growth parameters (fresh and dry weight, biomass yield, and productivity) in suspension cultures of *T. officinale* maintained at different sucrose concentrations are presented. Sucrose concentration in the medium had a significant effect on cell growth (*p* < 0.01). Suspension cultures maintained at 2.3% (*w*/*v*) of sucrose showed the highest value of fresh biomass (Figure 4a) with a value of 204.4 g/L, followed by the cultures maintained at 1.0%, 3.2%, and 5.5% (*w*/*v*) of sucrose, with values of 136, 83, and 42 g/L, respectively. In terms of dry weight, it was largest in the suspension medium at 2.3% (*w*/*v*) of sucrose, showing a value of 12 g dw/L (Figure 4b), followed by the cultures maintained at 1.0%, 3.2%, and 5.5% (*w*/*v*) of sucrose, with values of 6.3, 5.6 and 3.3 g/L, respectively.

Biomass yield on the carbon source (Y_X/S_) was similar for all the conditions tested. Y_X/S_ values of 223, 237, 236, and 257 mg/g were obtained for suspension cultures maintained at 1.0%, 2.3%, 3.2%, and 5.5% (*w*/*v*), respectively (Y_X/S_ was calculated with Equation (4), see methods). Thus, carbon source concentration was not statistically significant (*p* = 0.68) on biomass yield, and it can be considered to have an average value of 238 mg/g. Cell productivity (Q_x_, in dw) values for cultures maintained at 1.0%, 2.3%, 3.2%, and 5.5% (*w*/*v*) of sucrose had values of 0.8, 1.5, 0.7, and 0.4 g/L-week, respectively (Q_x_ was calculated with Equation (5), see methods). Statistically, the analysis indicated that the effect of sucrose concentration was significant on cell productivity.

**Figure 5:**
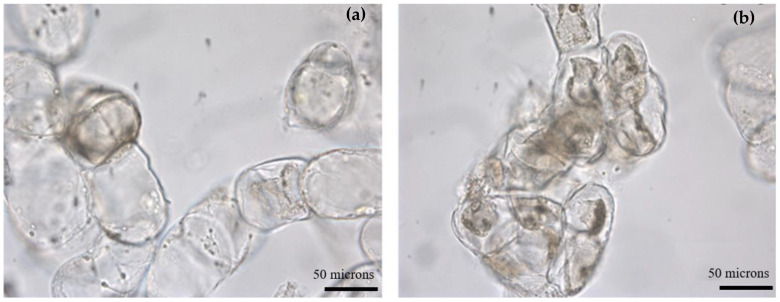
Cell clusters in suspension cultures of *T. officinale* maintained at (**a**) 1.0% (*w*/*v*) sucrose, (**b**) 5.5% (*w*/*v*) sucrose (Basal MS medium, 3.0 mg/L NAA, 3.0 mg/L BAP, 20 ± 2 °C, 115 rpm, 8th week of culture (bar = 50 microns).

In Figure 5a,b, suspension cultures of *T. officinale* maintained at 1.0% (*w*/*v*) and 5.5% (*w*/*v*) of sucrose are shown, respectively. At the lower carbon source concentrations, a predominance of rounded cells fulfilled with the cellular content were observed, while at 5.5% (*w*/*v*) most of the cells presented a condensed cellular content and were surrounded by a considerable amount of debris. However, contrary to what we expected based on medium osmolality, the humidity content of the cells was not significantly affected by sucrose content (*p* > 0.05), but a slight decrease in the average value as the sucrose concentration increased was observed.

#### 2.2.2. Effect of Carbon Source on Triterpenes Content

**Figure 6:**
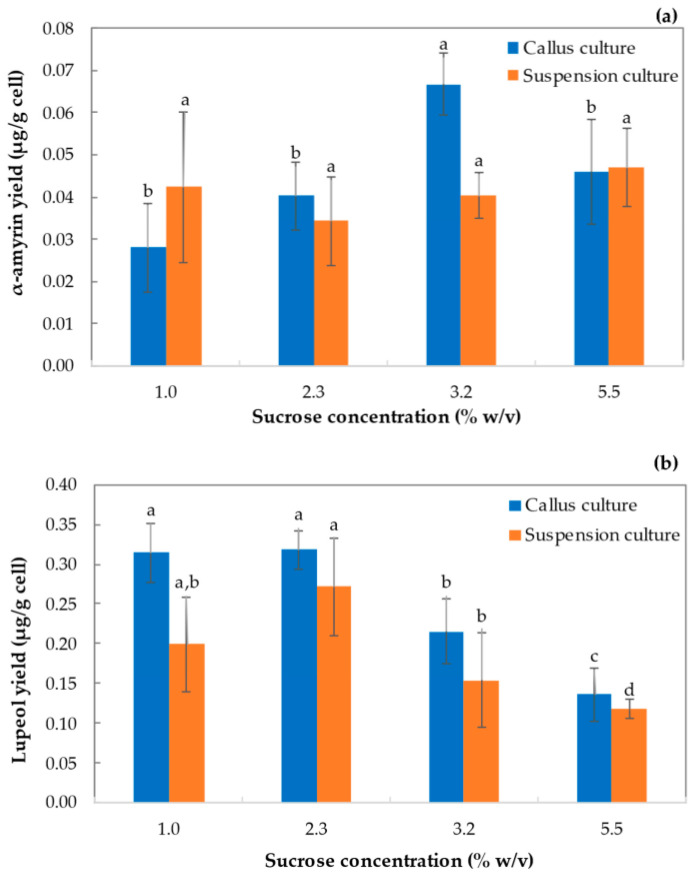
Effect of carbon source on triterpenes content. (**a**) α-Amyrin and (**b**) lupeol biomass yield in callus and suspension cultures of *T. officinale*. (Basal MS medium, sucrose 1.0%, 2.3%, 3.2%, and 5.5% (*w*/*v*), 3.0 mg/L NAA, 3.0 mg/L BAP, pH 5.8, 18 ± 2 °C, 0/24 h light/dark. 8th week of culture). Means with the same letter above the bars were not significantly different at the 0.05 level according to DMRT test. Bars indicate standard error.

In general, sucrose concentration in the growth medium was significant on lupeol yields in the callus (0.318 mg/g dw cell; *p* < 0.01) and suspension cultures (0.272 mg/g dw cell; *p* < 0.05). However, this parameter did not show a significant effect on α-amyrin accumulation (*p* > 0.5) in cell suspension (Figure 6a,b) (Figures S2 and S3, Supplementary material).

For the callus culture, after 8 weeks, the α-amyrin yield on cell (Y_ami/X_) (Figure 6a) was similar with an average yield of 0.038 mg/g under the different sucrose conditions except for sucrose 3.2% (*w*/*v*), that presented a yield of 0.067 mg/g. For the suspension culture, yields were similar to those obtained for the callus culture (*p* > 0.05) without being affected by the carbon source concentration in the liquid media. An average value of 0.041 mg/g was obtained. Regarding lupeol (Figure 6b), the highest content of this triterpene was obtained in the callus culture supplemented with sucrose 1.0% and 2.3% (*w*/*v*), presenting yields of 0.314 and 0.318 mg/g, respectively. At sucrose 3.2% and 5.5% (*w*/*v*), Y _lup/X_ values were 0.215 and 0.136 mg/g, respectively. For the suspension cultures maintained in medium supplemented with 1.0%, 2.3%, 3.2%, and 5.5% (*w*/*v*), Y_lup/X_ values were 0.199, 0.272, 0.154, and 0.118 mg/g, respectively. In the suspension cultures, triterpenes yields were up to 40% lower for lupeol and 50% lower for α-amyrin compared to the callus culture.

**Figure 7:**
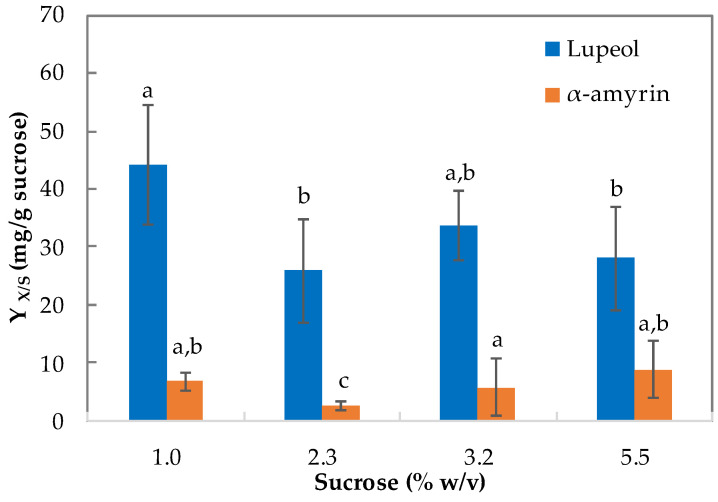
Yields (on substrate) for α-amyrin and lupeol in suspension cultures of *T. officinale*. (Basal MS medium, sucrose 1.0%, 2.3%, 3.2%, and 5.5% (*w*/*v*), 3.0 mg/L NAA, 3.0 mg/L BAP, pH 5.8, 18 ± 2 °C, 0/24 h light/dark. 8th week of culture). Means with the same letter above the bars were not significantly different at the 0.05 level according to the DMRT test. Bars indicate standard error.

In terms of the product yield on the carbon source (only measured for the suspension cultures) (Figure 7), sucrose concentration has a weak effect (*p* = 0.49) on lupeol yield (Y _lup/S_), for which values ranged from 28 to 44 mg/g, minimum at 2.3% (*w*/*v*) and maximum at 1.0% (*w*/*v*). However, an average value of 31 mg/g was calculated. Sucrose did not have a statistically significant difference on α-amyrin (Yami/S) (*p* > 0.05) when the medium was supplemented with 1.0%, 3.2%, and 5.5% (*w*/*v*) sucrose, for which a yield of 7.0 mg/g was calculated. However, the lowest α-amyrin yield was at 2.3% (*w*/*v*) sucrose.

## 3. Discussion

### 3.1. Effect of Inoculum Age and Size on Cell Suspension Culture Establishment

The inoculum, that primarily derives from callus cultures, is also a determinant for a proper suspension culture initiation. Callus culture is maintained for extended periods by subculturing, representing a convenient way of long-term maintenance of a specific cell line [27]. In this sense, proper maintenance conditions and the quality of the callus used as the inoculum have an important effect on the performance of the suspension culture and may be critical for the success of its establishment [27,29]. Specifically, inoculum size and age are extremely important variables because it has been reported that some compounds are secreted from the callus to the medium stimulating their growth, needing a minimum amount of callus to resume the growth in a new culture. However, other authors report that a large inoculum may not be adequate because it produces “staling” compounds that affect growth [30] or that it might cause a fast nutrient depletion in the media [31]. The minimum number of cells needed to establish a suspension culture might be closely related to species and culturing conditions. However, having better growth at certain inoculum densities indicated that cell growth requires a certain initial density of cells up to an optimum concentration, and that a lower inoculum size might be inhibitory to the growth of suspension cultures [32]. It has been widely reported that low inoculum concentrations induce longer lag phases for plant cell cultures, and when a culture is transferred to a fresh medium, key growth factors diffuse out of the cells to the surrounding medium [29]. Low densities in the medium are detrimental to cell growth during this adaptation period, in which the cells cannot equal the rate of duplication to the rate of death during the initial adaptation period [33].

The inoculum’s age and size were found to be significant on cell suspension establishment and cell growth, in which suspension cultures initiated using a 6-week-old callus gave the highest growth rate (see Figure 1a,b). During cell growth, metabolism changes towards the adaptation to the environment, and the growth rate is determined by metabolism. A younger culture (growing in the lag phase) is still adapting to the culture conditions from the callus initiation, and cells present a lower growth rate. Therefore, these cells might not be capable of multiplying at a rate greater than cell death or not be suitable for a rapid response to the new environment. On the other hand, the accumulation of necrotic debris and toxic compounds from the callus culture can also be detrimental to suspension culture [34,35,36,37]. This might be why a higher percentage of the cells died within the first four weeks (or two subcultures). During this period, the callus was probably adapting to the new culture system, transitioning from a solid medium to being entirely immersed in liquid. This adaptation period also affected the viability of the cells. In the beginning, cells were mechanically detached from the callus clumps but they would not necessarily be viable and multiplicative. The lower growth achieved with cultures older than 6 weeks was probably due to the change of the cells to a new physiological state rather than nutrient or oxygen depletion. This environmental change could be also the reason why growth was observed as slower under suspension conditions than observed for callus culture, as previously reported for *T. officinale* [38]. It has been stated that cell growth is faster in suspension cultures because cells are in direct contact with the nutrients and gases, allowing rapid mass and gas transferences. However, in all cases, the suspension cultures of *T. officinale* grew slower than the respective callus culture.

To the best of our knowledge, the effect of callus age and size has not been studied for the *Taraxacum* genus on suspension culture establishment. The effect of inoculum age on the establishment has been studied for several species. For *J. curcas* cell suspensions, the use of 60-day-old calli had a negative effect on growth (as fresh and dry weight), as lower values were observed for cell biomass compared to the use of 35-day-old calli [13]. In another study, suspension cultures of *C. canephora* initiated using 28- and 35-day-old callus had the best growth performance in rate and morphology, while a decrease in growth occurred with increased calli age [36]. These results agree with those obtained in this work, in which 6-week-old (42 days-old) calli showed the best results for suspension culture initiation. From the 4th to the 8th week of culture (during the 2nd and 4th subculture), *T. officinale* callus was growing rapidly (presumably in its exponential phase), allowing for its use as an inoculum to maintain an accelerated growth. However, when using an inoculum in the sixth week (3rd subculture), higher viability was observed (see Figure 1d). In terms of inoculum size, authors have reported that the use of less than 1% (*w*/*v*) of callus for *Vitis vinifera* suspension cultures could not initiate cell division and proliferation, while cell suspensions turned brown with a dramatic decrease of growth when initiated with 2% (*w*/*v*) of callus [33]. In another study, authors reported that the growth of *Artemisia annua* cell cultures was inversely proportional to the initial inoculum, with maximum growth attained using an inoculum density of 1% (*w*/*v*), and that the cultures with higher initial inoculums seemed to show limited growth when compared with cultures with lower initial inoculums. This is likely because exhaustion of oxygen or depletion of nutrients in the culture medium occurred at the beginning of the culture period due to the excess of inoculum [37]. This effect was also indicated for *Cyperus aromaticus* suspension cultures initiated using different inoculum sizes (0.3%, 1%, 1.6%, 3.3%, and 5% (*w*/*v*)) [39], in which the authors observed that using above 1% *w*/*v* of inoculum size might cause nutrient depletion in the media. In another study on *Pogostemon cablin* cell suspension cultures, growth was higher in cultures initiated with 10% (*w*/*v*) [31]. Therefore, a higher initial inoculum did not directly produce greater cell biomass. The best results on biomass growth found in the literature for suspension cultures were similar to those obtained in this work, using an inoculum size of 4% (*w*/*v*).

As observed in our experiments (data not shown), debris has been also reported for suspension cultures of *Coleus forskohlii* and *Catharanthus roseus*, in which debris (such as pieces of the cell wall, clumped cytoplasmic materials, starch granules, etc.) could be due to inadequate inoculum densities [40,41]. However, periodical change into a new medium and the filtration of the suspension culture maintained a predominance of small (Group 1, <5 cells/aggregate) and medium clusters (Group 2, 6–25 cells/aggregate) during the experimental period. Specifically, small clusters (Group 1) were observed profusely in the suspension cultures, primarily at the beginning of the cultures. However, periodical filtering prevented the accumulation of larger aggregates. It has been stated that during the period of most active cell division, the suspensions normally show maximum aggregation but are not essential for high rates of growth and division in cell suspension cultures. The subculturing of the cultures maintained the cells in an actively dividing state, minimizing cell aggregation, probably because of the mechanical forces and continuous filtering. The importance of maintaining a homogeneous suspension culture is because the lack of uniformity in suspension cultures can affect cell growth. Aggregation occurs when daughter cells fail to separate after cell division, promoted by extracellular polysaccharides, and varying between cell lines, the age of the cells, and the growth conditions [42].

### 3.2. Effect of Inoculum Age and Size on Triterpene Content

Regarding triterpene content, both α-amyrin and lupeol were detected in the callus when inoculum age and size were above 2 weeks and 2% (*w*/*v*), respectively. The identification of these two compounds agrees with a previous work, which used leaf extracts from the wild plant, in which lupeol acetate, lupeol, α-amyrin, β-sitosterol, and betulin (among others) were identified in hexane and ethyl acetate extracts. In 2018, Díaz et al. [28] indicated that lupeol content (23.31%) was higher than α-amyrin (4.78%) in wild plants as opposed to in vitro cultivated plants. Also, in this study the same trend was detected in dandelion cell suspension culture under in vitro conditions, with an average amount of lupeol (31 mg/g) higher than that of amyrin (6.5 mg/g), regardless of the concentration of the carbon source (sucrose) evaluated (Figure 7). The effect of inoculum characteristics has been studied previously in berberine production from *Tinospora cordifolia* Miers suspension cultures, in which cell aggregate size impacted mass transfer effects that might govern the metabolite synthesis. The authors reported that cell aggregates of 500 µm in diameter promoted the production of biomass, but larger cell aggregates up to 2000 µm in diameter favored berberine accumulation [43]. Maximum triterpene accumulation was obtained with an 8-week-old inoculum, while maximum growth was obtained with a 6-week-old inoculum. Interestingly, suspension cultures of *Ficus deltoidea* showed that the highest production of biomass was obtained from an initial inoculum size of 8% (*w*/*v*), whereas the highest flavonoid was found when the inoculum was 2% (*w*/*v*) of media [44]. Therefore, a decoupling of triterpene accumulation with cell growth can be supposed. Nevertheless, solasodine productivity was achieved using larger (20% (*w*/*v*)) rather than smaller (10% *w*/*v*) inoculum corresponding with higher dry cell weight [45]. Thus, a new set of experiments for studying α-amyrin and lupeol accumulation during cell growth to set the best compound production strategy should be proposed.

### 3.3. Effect of Sucrose Concentration on Cell Growth in Suspension Culture

Best growth in fresh and dry weight, and therefore cell productivity, was obtained when the cells were grown in media supplemented with sucrose 2.3% (*w*/*v*) (see Figure 3 and Figure 4a,b,d). Biomass yield on sucrose consumption was almost constant despite the sucrose concentration in the medium slightly increasing at 5.5% (*w*/*v*) (see Figure 4c). Humidity slightly decreased when the carbon source decreased in the medium. It has been stated that the accumulation of starch in the cells as a result of higher osmotic pressure can be present during suspension cultures [46]. Values obtained here were similar to those reported for other suspension cultures, reporting values of 0.18 g dw/g for *Elaeis guineensis* [32] and 0.4 g dw/g for *Dioscorea deltoidea* [47].

Differing outcomes have been reported regarding the effects of sucrose concentration on plant cell cultures. In cell suspension cultures of *Melastoma malabathricum*, 1.5% (*w*/*v*) sucrose led to the highest fresh cell weight, while the highest dried cell weight was obtained from the cells cultured in the medium supplemented with 45 g/L of sucrose. However, when the sucrose level was increased to 6.0% (*w*/*v*), there was a decreasing trend in cell growth [48]. In another work, sucrose at 6.0% (*w*/*v*) allowed a higher dry matter accumulation when compared to lower sucrose concentrations, even when the lower level of fresh biomass was probably caused by an osmotic effect [49]. On *Perilla frutescens* cell cultures, growth rates increased with the increase in initial sucrose concentration from 1.5% to 6.0% w/v in the medium, suggesting that, in a medium in which all nutrients were present in excess, an increase in sugar concentration could result in a proportional increase in cell biomass [50]. Meanwhile, in suspension cultures of *J. curcas*, growth at different sucrose concentrations was higher when the culture medium was supplemented with 3.0% and 4.0% (*w*/*v*) of sucrose, and growth decreased when sucrose concentration was reduced to less than 2.0% (*w*/*v*) [13]. On the contrary, sucrose at 3.0% (*w*/*v*) and 5.0% (*w*/*v*) resulted in a 3.9-fold and a 3.3-fold increase in growth during *Artemisia absinthium* L cultures, respectively [22], while stimulatory effect of sucrose concentration up to 5.0% (*w*/*v*) was observed on biomass accumulation in cell cultures of *Taxus chinensis* [51], *Ocimum sanctum* [52], and *Glycyrrhiza inflate* [53]. On the contrary, inhibition in biomass accumulation in response to sucrose concentrations higher than 30 g/L was observed in suspension cultures of *Prunella vulgaris* [54]. These reports are somewhat in line with the results found in this study, where better growth and quality of the cells were obtained in culture media supplemented with 1.0% and 2.3% *w*/*v* of sucrose.

Sucrose concentration was significant on lupeol yields in the callus and suspension cultures, though this parameter did not show a significant effect on α-amyrin accumulation (see Figure 6). Moreover, triterpene accumulation in suspension cultures was lower (up to 30%) than in the callus culture at similar conditions, especially for lupeol. Results indicated that the accumulation of these compounds on suspension cultures was lower than reported for other cultures, and even lower in comparison with the respective callus cultures. For instance, the alkaloid content and yield were three times higher in the suspension culture of *Catharanthus roseus* L. when compared to a solid medium under similar treatments [55]. Overall, α-amyrin and lupeol concentrations and yields were considerably lower than those reported in the literature for other triterpenes recovered from cell cultures. α-Amyrin and lupeol were lower than 0.1 mg/g dw and 0.5 mg/g dw, respectively, for all the conditions tested. These values are considerably lower compared to those reported previously. For instance, *T. officinale* callus culture accumulated triterpene acids and triterpenol accounting for 0.11%, 0.07%, and 0.29% dw for triterpenes, triterpenols, and triterpenes esters, respectively [24]. However, in this work, no triterpene acids and esters were measured that could be of great importance in future research to complement these results. Furuno et al. [23]. indicated that triterpenols were minor in callus cultures when compared to triterpenic acids. In callus cultures, the authors obtained around 0.5 mg/g dw of triterpenols (similar to that obtained here), while between 2.0 and 8.0 mg/g dw were contained in shoots and roots of plants. Moreover, even higher concentrations of triterpenes were reported in wild plants, with up to 25% of β-amyrin and 4.0% of sitosterol in the leaves and roots of *T. officinale* [56,57]. These results suggest that in vitro culture of undifferentiated cells of *T. officinale* should be useful to produce triterpenic acids rather than triterpenols.

Reports of higher values for similar triterpenoids include suspensions of *Lantana camara* L., in which 2.2% (*w*/*v*) of betulinic, oleanic, and ursolic acids were achieved [58]. Another report indicated that ursolic and oleanolic acids accumulated up to 2.1 mg/g dw in cell cultures of *Uncaria tormentosa* [59]. However, lower triterpene accumulation was reported for the same species (up to 0.6 mg/g dw in cell cultures [60]). Compound yields and biomass production are extremely important in order to achieve the highest possible production of bioactive compounds. It appears, therefore, that screening cell lines for high and stable production of secondary metabolites in cultures is an important factor for commercial exploitation [61]. For instance, [62], screened hundreds of *Plumbago rosea* L. lines for plumbagin accumulation in suspension cultures solely to find a few highly promising lines.

In this study, lupeol concentration was up to three times higher than the α-amyrin concentration at every condition, including the leaves of the in vitro plants from which explants were derived. Authors reported higher concentrations of triterpene esters in wild shoots and roots than in callus cultures, while triterpene acids in wild shoots were not identified [24]. This means that the accumulation of the different kinds of triterpenoids is likely tissue-specific and that some degree of differentiation for the accumulation of certain types of compounds can be expected, in which undifferentiated tissue culture seems not completely suitable (in this case, probably α-amyrin).

No information was found on triterpenes yield on sucrose or any carbon source for the studied triterpenes. To the best of our knowledge, sucrose concentration has not been studied in relation to α-amyrin and lupeol synthesis for the *Taraxacum* genus. The effect of the carbon source was found to depend on the specific compound and the plant species. For instance, increasing sucrose from 2.0% to 5.0% (*w*/*v*) in *Uncaria tomentosa* cell suspension cultures enhanced ursolic acid and oleanolic acid production from 0.13 to 0.55 mg/g dw cell [60]. Rosmarinic acid accumulation was largest in cultures supplemented with 4.5% (*w*/*v*), followed by the concentrations obtained at 6.0% (*w*/*v*) [49]. Moreover, sucrose also enhanced the production of other secondary metabolites such as diosgenin in *Dioscorea deltoidea* [47] and anthraquinone in *Galtium mollugo* [63]. Moreover, an increase of initial sucrose concentration above the normally used level (3.0% *w*/*v*) enhanced the accumulation of flavonoids in cell cultures of *Glycyrrhiza inflata* and callus cultures of *Eryngium planum* L. [64], saponin and polysaccharide content in cell cultures of *Panax ginseng* [49], and taxol production in cell cultures of *T. brevifolia* [65]. In contrast, Baque et al. [66] have reported sucrose 1.0% (*w*/*v*) as the optimum concentration for the maximum accumulation of anthraquinone, phenolics, and flavonoids in *Morinda citrifolia*. Sucrose at 3.0% (*w/v*) was beneficial in the production of ajamalicine and catharanthine from immobilized cells of *C. roseus* using a conditioned medium [67]. However, sucrose ranging from 2.0% to 6.0% (*w*/*v*) increases the production of arbutin from suspension cultures of *C. roseus* by glucosylation of exogenous hydroquinone [68]. Maximum accumulation of betacyanin in the suspension culture of *Phytolacca americana* was enhanced by increasing the number of cells in the presence of 88 mM sucrose and by fresh weight in 175 mM sucrose-containing medium [69]. In another report, authors tested different sucrose concentrations to enhance cell growth and production of the phytomedicinal compound zerumbone in suspension cultures of *Zingiber zerumbet* Smith, showing that the production of this compound was not significantly affected by different concentrations of sucrose between 1.0% and 3.0% (*w*/*v*) [70]. These dissimilar results gathered from the literature suggest differing responses of cell lines of diverse species to the same culturing conditions, and therefore, the results must be taken as guidance. Nevertheless, it is somehow suggested that lower carbon source concentrations can improve biomass proliferation while secondary compounds accumulation can be improved by higher concentrations. Therefore, a two-stage strategy for growth and triterpene accumulation in suspension cultures of *T. officinale* can be considered for subsequent experiments.

Through the course of the experiments, it was observed that, at the beginning of the cultures, the cells presented spherical shapes, likely derived directly from the surface of the callus aggregates. Yellow callus showed predominantly small and isodiametric cells at the beginning of the experiment. In older cultures, cells were elongated and consist of chains of different lengths, likely due to cell division and differentiation. It has been stated that the embryogenic state is characterized by small densely cytoplasmic cells, while suspensor cells are highly vacuolated and elongated [71]. However, during the entire experimental period, suspension cultures were a mixture of different cell morphologies. No organogenesis or other structures appeared in the suspension culture during this experience, probably because potential embryos that could be developed were removed by periodic filtration. Moreover, the continuous supply of equal amounts of NAA and BAP (3.0 mg/L each, in combination) probably maintained low organogenesis in the callus culture of *T. officinale*, as seen in previous experiments [38].

## 4. Materials and Methods

Experiments were carried out at the Nucleus of Biotechnology of Curauma (NBC), the Pontifical Catholic University of Valparaiso (PUCV), and the Centre of Systems Biotechnology-Fraunhofer Chile Research (FCR-CSB) in Chile.

### 4.1. Callus Culture

Callus induction from *T. officinale* hypocotyl of 1 month-old, sterile, in vitro cultivated plants in 4.4 g/L Murashige and Skoog medium basal including vitamins (MS) (PhytoTech Labs, Lenexa, KS, USA) [72], 2.3% (*w*/*v*) of sucrose and 7 g/L agar (Duchefa, The Netherlands), pH 5.6 and supplemented to 0.5 mg/L of NAA and 0.5 mg/L of BAP.

Cultures were maintained in complete darkness in the culture room at 22 ± 2 °C in 100 mm-diameter Petri dishes containing 20 mL of medium sealed with parafilm “M”^®^. For callus maintenance medium (CM), the same preparation mentioned above was used, but with the addition of 3.0 mg/L NAA and 3.0 mg/L BAP, as conditions that promote cell proliferation in good quality [38].

### 4.2. Cell Suspension Culture

Cell suspension culture was initially established by inoculating a white-friable callus into a 125 mL Erlenmeyer conical flask containing 10 mL CM medium as indicated in Section 4.2.1, without agar addition. Flasks were covered with cotton wool and aluminum foil and placed on an orbital shaker at 115 rpm under darkness at 22 (±2) °C. After four weeks of culture initiation, cells were harvested from the liquid medium by filtering through a sterilized stainless-steel sieve with 1.0 mm pores to separate small cell aggregates from bigger clusters. After the clusters were removed, the cell suspension was centrifuged at 1000× *g* rpm to collect the cells, and an aliquot (5.0 mL) of the cell-containing concentrated medium was transferred into 10 mL of fresh medium and subculturing every 2 weeks for a total period of 12 weeks.

To ensure the correct cell growth in the suspension culture, the filtrate was placed on a slide for microscopic observation, in which differentiation, aggregation, and viability were monitored at every subculture. Medium pH was also monitored every week.

#### 4.2.1. Experiment 1—Effect of Inoculum Age and Size on Cell Growth in Suspension Culture Establishment

White-friable callus of different culture ages (2 to 10 weeks) and sizes (0.2–8% (*w*/*v*)) were used as inoculum and transferred into 125 mL Erlenmeyer flasks containing CM medium for suspension culture establishment by following the protocol mentioned in Section 4.2. A Response Surface Model (RSM) (see Section 4.4) was evaluated between the age and size as variables (Table 5) in a 2FI Model (a two-factor-interaction model). A central composite design was applied to optimize the response of each factor considered (Table 6). At the end of the culturing period, flasks of each treatment were taken and cell growth (fresh and dry weight) and triterpene (α-amyrin and lupeol) content were measured. Each treatment consisted of three replicates. In addition, cell viability, aggregation, and differentiation were determined.

An RSM was used as an experimental optimization design. Two response surface factors were selected, and the range of values X_1_ (Age, 2–10 weeks), and X_2_ (Size, 0.2–8% (*w*/*v*)) was determined based on preliminary experiments (Table 5). The coded values for the design are “1” for maximum value and “−1” for minimum value, covering a fractional factorial design and providing a total of 19 treatments.

Once inoculum conditions (callus age and size) were selected using RSM performed in experiment 1, the stock culture of callus was maintained in MS medium supplemented with 2.3% (*w*/*v*) sucrose for experiment 2.

#### 4.2.2. Experiment 2—Effect of Carbon Source on Triterpene Accumulation in Suspension Culture Establishment

From stock culture, media supplemented with different sucrose concentrations were inoculated for triterpene (α-amyrin and lupeol) content in suspension cultures. CM medium was modified in its sucrose concentration, for which 1.0%, 2.3%, 3.2%, and 5.5% (*w*/*v*) of this sugar were evaluated.

Samples for determining cell growth and quantification of terpenes were taken every week during the 12 weeks of the experiment; three flasks of each treatment were taken and cell growth (fresh and dry weight) and triterpene (α-amyrin and lupeol) content were measured (see Section 4.3.2). In addition, cell viability, aggregation, and differentiation were determined.

For comparison of triterpene content, a callus culture on solid CM medium under the same operational conditions was assessed.

### 4.3. Parameters Evaluated in Experiment 1 and 2

#### 4.3.1. Cell Growth Measurements

##### Fresh Weight (fw), Dry Weight (dw)

These parameters were gravimetrically measured. For the callus culture, cell masses were collected and weight directly on the scale. For suspension cultures, samples collected were filtered through a Whatman®, grade N°1 paper filter. After the determination of the fresh weight, cells from solid or liquid cultures were dried at 50 °C for 24 h in an oven and weighed. Humidity was considered as the cell weight loss after oven drying. These data were used for growth kinetics and yield calculation.

##### Carbon Source

The sucrose concentration of the growth medium was determined according to the method proposed by Dubois et al. [73], with some minor modifications. Sucrose remaining in the suspension culture was quantified during each subculturing after cells were harvested. These data were used for yield calculations.

##### Viability, Differentiation, and Aggregation

At every subculture, a 20 μL aliquot of each suspension culture was observed under the microscope. Viability was determined as the percentage of living cells (with no evident cellular damage) to the total number of cells observed within each frame (a total of 20 frames) and by the trypan blue staining test [74]. Differentiation was monitored by observing cell wall structures and organ formation. Aggregation was monitored through microscopic observation (Microscope: Olympus CX31, Olympus Corporation, Japan) and evaluated by grouping the cell clusters as observed within each frame. Clusters were grouped as follows: Group 1 (small cluster) < 5 cells/aggregate; Group 2 (medium cluster) 6–25 cells/aggregate; and Group 3 (large cluster) > 25 cells/aggregate.

#### 4.3.2. Quantification of α-Amyrin and Lupeol Content

##### Cell Extraction

For the callus culture, calli were taken directly from the plates and lyophilized at −40 °C and 560 psi for 8 h. Two grams of freeze-dried calli were ground in a porcelain mortar, placed in centrifuge tubes with 20 mL of methanol:chloroform (1:1), and stirred for 30 min. From the suspension culture, 50 mL was taken and centrifuged (2000 rpm× *g* for 5 min) for cell recovery. The culture was vacuum filtered on Whatman No. 1 paper. Cells were lyophilized (Virtis Company Benchtop Lyophilizer, New York, NY, USA) and extracted in Eppendorf tubes with 1.0 mL of methanol:chloroform (1:1) and stirred for 30 min. The residue of the extraction was removed by filtration using a syringe with 0.45 and 0.22 μm nylon filters (Millipore). The remaining solvent was evaporated under vacuum at 40 °C. The dried extracts were resuspended in 1.0 mL of methanol and stored frozen (−20 °C) until further analysis.

##### α-Amyrin and Lupeol Reference Standards

α-amyrin (purity 99.3%) and lupeol (purity 99.7%) were purchased from Sigma-Aldrich Chemie GmbH (Aldrich Division, Steinbeim, Germany). Standard solutions of both compounds were prepared by adding 1.0 mg of the standard (α-amyrin or lupeol) to 1.0 mL methanol in a 1.5 mL Eppendorf tube. The tube was stirred for 2 min until the complete dissolution of the standard and stored frozen (−20 °C) until analysis was carried out (Figure S2a,b; supplementary material).

##### α-Amyrin and Lupeol Quantification

Identification and quantification of α-amyrin and lupeol was performed using the methodology of Adhyapak and Dighe [75] with some modifications, using high-performance liquid chromatography with a diode-array detector (HPLC-DAD; DIONEX Ultimate 3000 RSCL Systems, Thermo Fischer Scientific Inc, Waltham, MA, USA), Zorbax^®^ HPLC Column phase C18 (octadecyl), with a 5µm particle size, L × I.D. 25 cm× 4.6 mm. The protocol was set in an isocratic elution gradient of 30:70 *v*/*v* methanol/acetonitrile (0.1% TFA) at 1.0 mL/min for 20 min with an injection volume of 10 μL. The absorption of the compounds was carried out at 210 nm. Lupeol and α-amyrin were quantified by comparing the peak area, the spectral patterns, and the retention time obtained for the samples against the appropriate standard. Calibration curves of the two standards (α-amyrin and lupeol) were obtained by plotting the obtained areas against their corresponding concentration.

#### 4.3.3. Growth Parameters

As the feeding regime was similar to a fed-batch process, kinetic parameters were calculated for each batch and adjusted subsequently through iteration of the entire process.

Cell mass yield coefficient (Y_X/S_, g/g—Equation (4)), cell productivity (Q_x_, g/L·d—Equation (5)), α-amyrin and lupeol yield coefficients on biomass (Y_ami/X_, Y_lup/X_, g/g—Equation (6)), triterpene yields on substrate (Y_ami/S_, Y_lup/S_, g/g—Equation (7)), and volumetric productivities (Q_ami_ and Q_lup_, mg/L—Equation (8)) were calculated as follows:

(4) $$Y_{\frac{X}{S}}=\frac{X-X_{0}}{\sum_{i}^{n}\left(S-S0\right)}$$

(5) $$Q_{x}=\frac{X}{V\cdot t}$$

(6) $$Y_{\frac{\mathrm{ami}}{X}}=\frac{C_{\mathrm{ami}}}{\left(X-X_{0}\right)};{\;Y}_{\frac{\mathrm{lup}}{X}}=\frac{C_{\mathrm{lup}}}{\left(X-X_{0}\right)}\;$$

(7) $$Y_{\frac{\mathrm{ami}}{S}}=\frac{C_{\mathrm{ami}}}{\sum_{i}^{n}\left(S_{0}-S\right)};{\;Y}_{\frac{\mathrm{lup}}{S}}=\frac{C_{\mathrm{ami}}}{\sum_{i}^{n}\left(S_{0}-S\right)}\;$$

(8) $$Q_{\mathrm{ami}}=Q_{X}\cdot Y_{\mathrm{ami}};{\;Q}_{\mathrm{lup}}=Q_{X}\cdot Y_{\mathrm{lup}}$$

where t_0_ and t are the initial and the total time (d) of the experiment, respectively; X_0_ and X are the cell concentration (g/L) at the beginning (at *t*_0_) and the end (at *t*) of the experiment, respectively; S_0_ and S are the sucrose (% (*w*/*v*)) at the beginning and the end of the experiment, respectively; i is the subculture number with n = 6 (3 months); and V is the suspension culture volume (L).

### 4.4. Data Analysis

Data were analyzed using SPSS software (version 16.0) in which ANOVA analysis was performed. The presented results represent the mean ± SE of three replicates per cycle. Means for groups in homogeneous subsets are displayed.

The response surface methodology (RSM) was selected as a statistical method for the design of the experiment [76] to optimize the number of experiments and determine the interactions between the variables.

Suspension culture responses were obtained by the design shown in Table 5, a mathematical model was developed, and optimal values were found. For RSM, the experimental data were fitted with a common second-order polynomial Equation (9):

(9) $$Y=\beta_{0}+\sum \beta_{i}X_{i}+\sum \beta_{\mathrm{ij}}X_{i}X_{j}+\sum \beta_{\mathrm{ii}}X_{\mathrm{ii}}{}^{2}{\;}_{}$$

where Y is the predicted response, β_0_ is a constant, β_i_ is the linear coefficient, β_ii_ is the quadratic coefficient, and β_ij_ is the interaction coefficient. The DOE was performed using Design of Expert 7.0.0 software, using the quadratic design model.

The software provided coefficient of determination (R^2^) and lack of fit data to validate the model through analysis of variance (ANOVA). The suitability of the polynomial model equation’s fit was tested by the R^2^, adjusted R^2^ (Adj R^2^), predicted R^2^ (Pred R^2^), coefficient of variation (CV), and adequate precision (Adeq Pres). The non-significant terms were removed to obtain a reduced model (*p* < 0.05). The fitted polynomial Equation was further expressed as response surface plots (2D) to visualize the relation between the response and independent variables.

## 5. Conclusions

This is the first study on the establishment of cell suspension cultures and quantification of triterpenes of *Taraxacum officinale* under different inoculum and sucrose conditions in vitro. Because of cell growth, cell quality, and α-amyrin and lupeol yields, the conditions selected for the suspension cultures were its initiation with 4% (*w*/*v*) of a 6-week-old inoculum. Among the sucrose concentrations, 2.3% (*w*/*v*) supported maximum biomass (in quantity and quality) for at least 8 weeks of culture; however, the accumulation of triterpenes was favored at a sucrose concentration of 1.0% (*w*/*v*). Results indicate that 0.045 (±0.017) and 0.19 (±0.02) mg/g of α-amyrin and lupeol can be obtained in suspension culture under these conditions, respectively, being 50% and 40% lower than that obtained in callus, respectively. Thus, the results of the present study form a background for future studies related to large-scale production of α-amyrin and lupeol *T. officinale*.

## Figures and Tables

**Table 1:** Analysis of variance (ANOVA) and determination coefficients for the quadratic nonlinear model for dry cell growth [Partial sum of squares—Type III].

Source	Sum of Squares	df	Mean Square	F-Value	*p*-Value Prob > F	
Model	89.01	5	17.80	56.59	<0.0001	Significant
A-Age	0.89	1	0.89	2.83	0.1166	
B-Size	39.63	1	39.63	125.97	<0.0001	
AB	0.16	1	0.16	0.52	0.4823	
A^2^	32.25	1	32.25	102.53	<0.0001	
B^2^	30.36	1	30.36	96.50	<0.0001	
Residual	4.09	13	0.31			
Lack of Fit	2.44	2	1.22	8.17	0.0067	Significant
Pure Error	1.65	11	0.15			
Cor Total *	93.10	18				
Model Summary						
Std. Dev.		0.56		R-Squared		0.9561
Mean		3.41		Adj R-Squared		0.9392
C.V. %		16.45		Pred R-Squared		0.9044
PRESS		8.90		Adeq Precision		20.034

* Cor Total: Corrected Total; C.V: coefficient of variation; Adj R-Squared: Adjusted R-Squared; Pred R-Squared: Predicted R-Squared; Adeq Precision: Adequate Precision; PRESS: predicted residual error sum of squares; Std. Dev: Standard deviation.

**Table 2:** Analysis of variance (ANOVA) and determination coefficients for the quadratic nonlinear model for fresh cell growth [Partial sum of squares—Type III].

Source	Sum of Squares	df	Mean Square	F-Value	*p*-Value Prob > F	
Model	1.121 × 10^5^	5	22,411.89	15.74	<0.0001	Significant
A-Age	671.11	1	671.11	0.47	0.5044	
B-Size	48,968.04	1	48,968.04	34.39	<0.0001	
AB	495.38	1	495.38	0.35	0.5654	
A^2^	44,830.03	1	44,830.03	31.49	<0.0001	
B^2^	27,587.53	1	27,587.53	19.38	0.0007	
Residual	18,509.61	13	1423.82			
Lack of Fit	3567.40	2	1783.70	1.31	0.3080	Non-significant
Pure Error	14,942.21	11	1358.38			
Cor Total *	1.306 × 10^5^	18				
Model summary						
Std. Dev.		37.73		R-Squared		0.9071
Mean		117.27		Adj R-Squared		0.8037
C.V. %		32.18		Pred R-Squared		0.7217
PRESS		36,331.66		Adeq Precision		10.205

* Cor Total: Corrected Total; C.V: coefficient of variation; Adj R-Squared: Adjusted R-Squared; Pred R-Squared: Predicted R-Squared; Adeq Precision: Adequate Precision; PRESS: predicted residual error sum of squares; Std. Dev: Standard deviation.

**Table 3:** Analysis of variance (ANOVA) and determination coefficients for the quadratic nonlinear model for viability [Partial sum of squares—Type III].

Source	Sum of Squares	df	Mean Square	F-Value	*p*-Value Prob > F	
Model	1133.71	5	226.74	25.39	<0.0001	1133.71
A-Age	45.15	1	45.15	5.06	0.0425	45.15
B-Size	682.27	1	682.27	76.40	<0.0001	682.27
AB	4.35	1	4.35	0.49	0.4975	4.35
A^2^	238.60	1	238.60	26.72	0.0002	238.60
B^2^	329.44	1	329.44	36.89	<0.0001	329.44
Residual	116.09	13	8.93			116.09
Lack of Fit	38.12	2	19.06	2.69	0.1120	38.12
Pure Error	77.98	11	7.09			77.98
Cor Total *	1249.81	18				1249.81
Model summary						
Std. Dev.		2.99		R-Squared		0.9071
Mean		86.85		Adj R-Squared		0.8714
C.V. %		3.44		Pred R-Squared		0.7642
PRESS		294.77		Adeq Precision		13.496

* Cor Total: Corrected Total; C.V: coefficient of variation; Adj R-Squared: Adjusted R-Squared; Pred R-Squared: Predicted R-Squared; Adeq Precision: Adequate Precision; PRESS: predicted residual error sum of squares; Std. Dev: Standard deviation.

**Table 4:** Quality parameters (viability and aggregation) for *T. officinale* suspension cultures maintained at different sucrose concentrations after an 8-wk culture.

Sucrose (% *w*/*v*)	Viability (%)		Aggregation Group 2 (6–25 Cells/Cluster) (%)		Differentiation (%)
**1.0**	87 ^a^	±3.6	59 ^a^	±4.1	ND
**2.3**	91 ^a^	±2.5	52 ^a^	±2.9	ND
**3.2**	87 ^a^	±2.8	41 ^b^	±3.4	ND
**5.5**	79 ^ba^	±5.1	28 ^c^	±1.9	ND

Means with the same letter down the group are not signifcantly different from each other. ND: Not detected under the microscope.

**Table 5:** Factors and levels of response surface method (RSM) analysis.

Independent Variables (Factor)	Variable Name	Levels Coded Values		Units
		−1	+1	
x_1_	Age	2	10	week
x_2_	Size	0.2	8	% *w*/*v*

**Table 6:** Fractional factorial design for the 2 independent variables (X_1_: inoculum size and X_2_: age).

Treatments	Independent Variables	
	x_1_ Age (wk)	x_2_ Size (% (*w*/*v*))
1	10	0.2
2	0.5	4.2
3	6	4.2
4	12	4.2
5	6	4.2
6	6	4.2
7	6	8.0
8	2	8.0
9	10	0.2
10	6	8.0
11	10	8.0
12	6	4.2
13	6	4.2
14	10	8.0
15	12	4.2
16	2	8.0
17	0.5	4.2
18	2	0.2
19	2	0.2

## Data Availability

The data sets generated and analyzed during the current study are 490 available from the corresponding author.

## Supplementary Materials

The following are available online at https://www.mdpi.com/article/10.3390/plants12051116/s1, Figure S1: pH Variation for *T. officinale* suspension cultures maintained at different sucrose concentrations for 8 total weeks of culture. Figure S2: Chromatogram of standards using a diode-array detector (HPLC-DAD; 210 nm; Ultimate 3000 HPLC, Thermo Fischer), Zorbax^®^ HPLC Column phase C18 (octadecyl), and 30:70 *v*/*v* methanol/acetonitrile (0.1% TFA): (a) Lupeol and (b) α-Amyrin (Adhyapak and Dighe, 2014 with modifications) [75]. Figure S3: Chromatogram (HPLC-DAD, 210 nm) *T. officinale* suspension cultures sample (Basal MS medium, sucrose 2.3 (*w*/*v*), 3.0 mg/L NAA, 3.0 mg/L BAP, pH 5.8, 18 ± 2 °C, 0/24 h light/dark. 8th week of culture).
