# Supplementary material for: Effect of Inoculum Size and Age, and Sucrose Concentration on Cell Growth to Promote Metabolites Production in Cultured Taraxacum officinale (Weber) Cells
Source: Plants (Basel). 2023 Mar 2;12(5):1116. doi: 10.3390/plants12051116 (PMC10004745; doi:10.3390/plants12051116)
Supplement: Supplementary file 1 [file plants-12-01116-s001.zip › plants-2006129-supplementary.pdf]

plants

MDPI

Article

Effect of Inoculum Size and Age, and Sucrose Concentration on Cell Growth to Promote Metabolites Production in Cultured *Taraxacum officinale* (Weber) Cells

María Eugenia Martínez [1], Lorena Jorquera [2], Paola Poirrier [1], Katy Díaz [3] and Rolando Chamy[1,*]

1 Escuela de Ingeniería Bioquímica, Facultad de Ingeniería, Pontificia Universidad Católica de Valparaíso, Brasil 2085, Valparaíso, 237463, Chile
2 Escuela de Ingeniería en Construcción y Transporte, Facultad de Ingeniería, Pontificia Universidad Católica de Valparaíso, Avenida Brasil 2147, Valparaíso 237463, Chile
3 Departamento de Química, Universidad Técnica Federico Santa María, Avenida España #1680, Valparaíso, 2390123, Chile
* Correspondence: katy.diaz@usm.cl (K.D.); rolando.chamy@pucv.cl (R.C.); Tel.: +56-32-2652843 (K.D.)

Citation: Martínez, M.E.; Jorquera, L.; Poirrier, P.; Díaz, K.; Chamy, R. Effect of Inoculum Size and Age, and Sucrose Concentration on Cell Growth to Promote Metabolites Production in Cultured *Taraxacum officinale* (Weber) Cells. *Plants* **2023**, *12*, 1116. https://doi.org/10.3390/plants12051116

Academic Editors: Laura Pistelli, Kalina Danova, Ilaria Marchioni and Iyyakkannu Sivanesan

Received: 29 November 2022
Revised: 17 February 2023
Accepted: 20 February 2023
Published: 2 March 2023

Copyright: © 2023 by the authors. Submitted for possible open access publication under the terms and conditions of the Creative Commons Attribution (CC BY) license (https://creativecommons.org/licenses/by/4.0/).

Supplementary Materials: The following are available online at www.mdpi.com/xxx/s1, Figure S1: pH Variation for *T. officinale* suspension cultures maintained at different sucrose concentrations for 8 total weeks of culture. Figure S2: Chromatogram of standards using a diode-array detector (HPLC-DAD; 210 nm; Ultimate 3000 HPLC, Thermo Fischer), Zorbax® HPLC Column phase C18 (octadecyl), and 30:70 *v/v* methanol/acetonitrile (0.1% TFA): (a) Lupeol and (b) α–Amyrin (Adhyapak and Dighe, 2014 with modifications) [75]. Figure S3: Chromatogram (HPLC-DAD, 210 nm*)* *T. officinale* suspension cultures sample (Basal MS medium, sucrose 2.3 (*w/v*), 3.0 mg/L NAA, 3.0 mg/L BAP, pH 5.8, 18 ± 2 °C, 0/24 h light/dark. 8th week of culture).

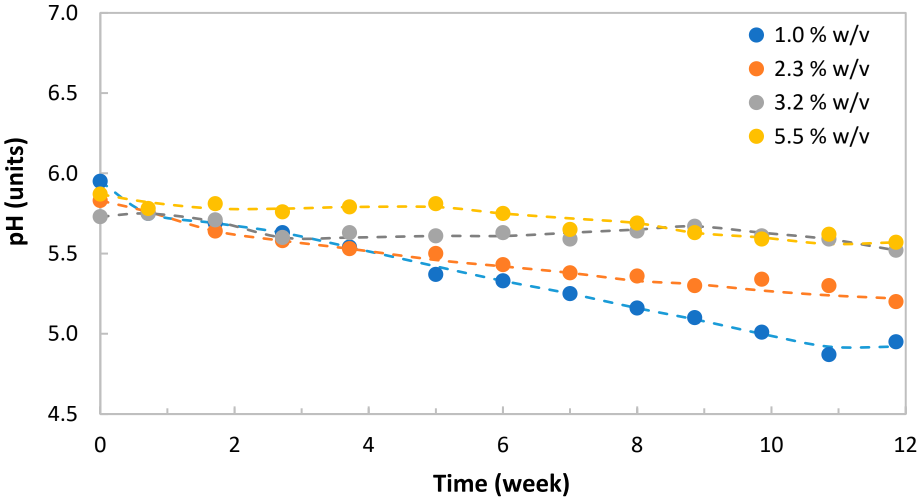

Figure S1. pH Variation for *T. officinale* suspension cultures maintained at different sucrose concentrations for 8 total weeks of culture.

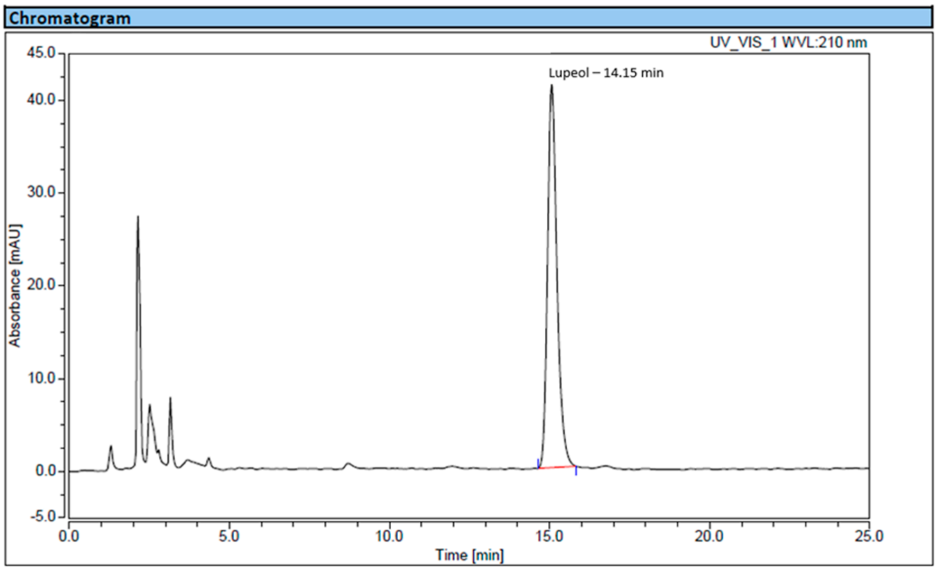

(**a**)

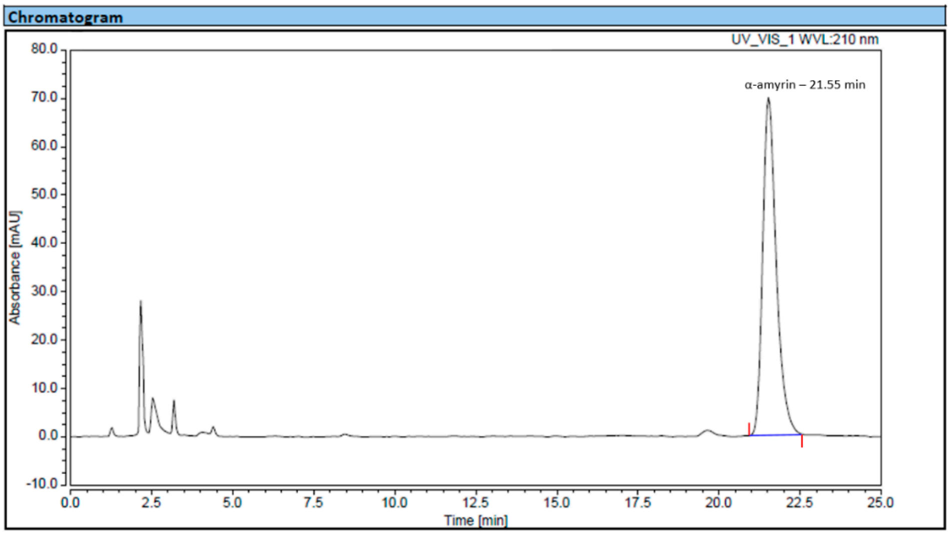

(**b**)

Figure S2. Chromatogram of standards using a diode-array detector (HPLC-DAD; 210 nm; Ultimate 3000 HPLC, Thermo Fischer), Zorbax® HPLC Column phase C18 (octadecyl), and 30:70 *v*/*v* methanol/acetonitrile (0.1% TFA): (**a**) Lupeol and (**b**) α–Amyrin (Adhyapak and Dighe, 2014 with modifications) [75].

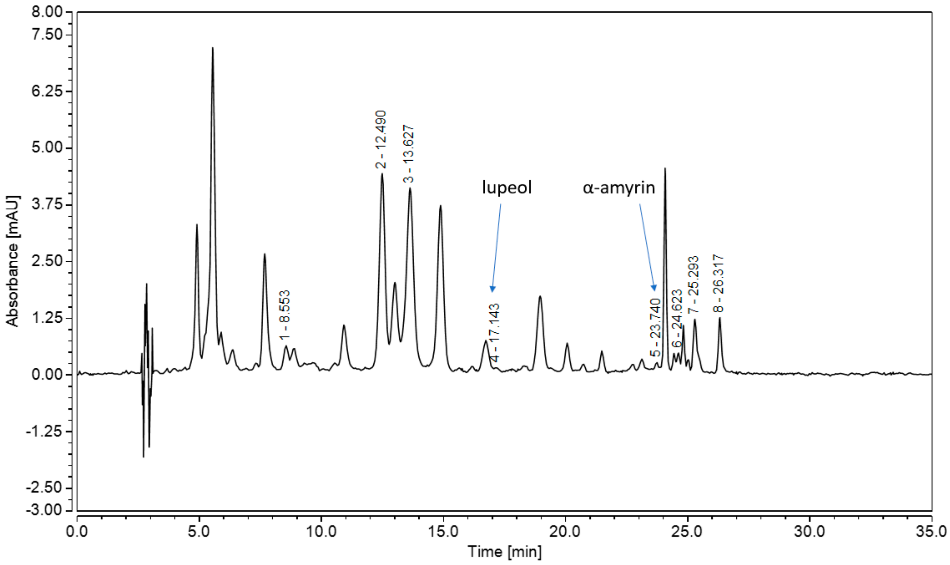

Figure S3. Chromatogram (HPLC-DAD, 210 nm) *T. officinale* suspension cultures sample (Basal MS medium, sucrose 2.3 (*w*/*v*), 3.0 mg/L NAA, 3.0 mg/L BAP, pH 5.8, 18 ± 2 °C, 0/24 h light/dark. 8th week of culture).
